# The Linker Pivot in Ci-VSP: The Key to Unlock Catalysis

**DOI:** 10.1371/journal.pone.0070272

**Published:** 2013-07-29

**Authors:** Kirstin Hobiger, Tillmann Utesch, Maria Andrea Mroginski, Guiscard Seebohm, Thomas Friedrich

**Affiliations:** 1 Institute of Chemistry, Max-Volmer-Laboratory of Biophysical Chemistry, Technische Universität Berlin, Berlin, Germany; 2 Department of Cardiovascular Medicine, Institute for Genetics of Heart Diseases, Myocellular Electrophysiology, University Hospital Münster, Münster, Germany; Dalhousie University, Canada

## Abstract

In the voltage-sensitive phosphatase Ci-VSP, conformational changes in the transmembrane voltage sensor domain (VSD) are transduced to the intracellular catalytic domain (CD) leading to its dephosphorylation activity against membrane-embedded phosphoinositides. The linker between both domains is proposed to be crucial for the VSD-CD coupling. With a combined approach of electrophysiological measurements on *Xenopus* oocytes and molecular dynamics simulations of a Ci-VSP model embedded in a lipid bilayer, we analyzed how conformational changes in the linker mediate the interaction between the CD and the activated VSD. In this way, we identified specific residues in the linker that interact with well-defined amino acids in one of the three loops forming the active site of the protein, named TI loop. With our results, we shed light into the early steps of the coupling process between the VSD and the CD, which are based on fine-tuned electrostatic and hydrophobic interactions between the linker, the membrane and the CD.

## Introduction

The different species of phosphoinositides play crucial roles in diverse signaling cascades [1]. Proteins that intervene in these pathways are of huge interest in medical research, because their insufficient functionality is associated with serious diseases, e.g. cancer [2] or neurodegenerative disorders like Alzheimer [3]. In this context, the *Ciona intestinalis* voltage sensitive phosphatase (Ci-VSP) is one of the model systems for investigating phosphoinositide-selective enzymatic catalysis on a molecular level.

In voltage sensitive phosphatases, the modular protein structure enables, in a unique manner, the direct transduction of an electrical signal across the plasma membrane into an intracellular chemical reaction.

**Figure 1:**
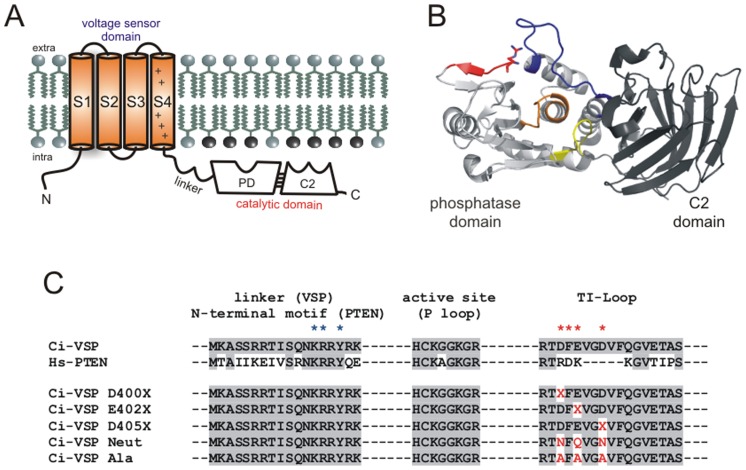
Topology scheme of Ci-VSP. *(* ***A*** *)* Topology scheme of Ci-VSP with the transmembrane voltage sensor (VSD) and the intracellular catalytic domain (CD), which comprises the phosphatase (PD) and the C2-domain. VSD and CD are connected via the linker sequence M240–K257. *(* ***B*** *)* Three-dimensional structure of the Ci-VSP CD based on the crystal structure by Liu et al. (PDB entry 3V0H) [14]. The P- (orange), the TI- (blue), and the WPD loop (yellow) form the active site of the CD. The linker (colored in red, with R253 shown as sticks) is oriented toward the TI loop, as proposed by Liu et al. [14]. *(* ***C*** *)* Amino acid alignment of Ci-VSP’s linker (M240–K257), active site (H362–R369), and the TI loop (R398–S414) with regions of the homolog PTEN (Hs, Homo sapiens). Amino acid identities are highlighted with a gray background. Putative interacting partners in the linker and the TI loop are denoted with blue and red asterisks, respectively. Single and multiple altered Ci-VSP mutants are aligned as well (in red letters: mutated positions). In the single neutralization mutants, the X stands for N (in case aspartate was the native residue) or for Q (in case of glutamate).

For this, a transmembrane voltage sensor domain (VSD) is coupled to a cytosolic catalytic domain (CD), which integrates a C2- and the enzymatic phosphatase domain (PD) (Fig. 1A, B). In Ci-VSP, the VSD-CD modules are coupled via a linker sequence of 18 amino acids (M240–K257, Fig. 1C).

Analogously to voltage-gated ion channels, the VSD consists of four putative α-helices, S1–S4, from which S4 contains several positively charged amino acids in a typical periodicity (Fig. 1A), which are responsible for voltage sensing of the protein [4].

The CD of Ci-VSP shares an overall amino acid homology of ∼40% to the human tumor suppressor PTEN (phosphatase and tensin homolog deleted on chromosome 10) [5], but the substrate specificities of both proteins are different. PTEN dephosphorylates membranous phosphoinositides selectively at the 3′-site position of the inositol ring [6], [7]. For Ci-VSP, a 3′-site selectivity was also demonstrated [8],[9]. However, its main substrate preference seems to be against the 5′-site position of phosphorylated phosphoinositides [8]–[10].

Since the discovery in 2005 [4], research on Ci-VSP has been focused on the intermodular coupling mechanisms that are responsible for the voltage-dependent activity of the protein. Upon depolarization of the membrane, conformational changes that occur in the VSD are transduced to the CD, which leads to the activation of the enzymatic domain [4], [10], [11]. The molecular processes that enable this VSD-CD interaction are dependent on the properties of the linker. Several studies revealed the importance of positively charged residues in the linker for electrostatic interactions with negatively charged membrane surfaces [11]–[13]. Thus, these interactions presumably lead to the binding of the linker to the membrane after the conformational change in the VSD has occurred. But what happens afterwards in the CD? Is the CD constitutively active, and does it just have to be recruited to the membrane in a diffusion-limited way? Or does it have to change its conformation during binding to the membrane-bound substrate? In both cases, the linker might be the crucial key in mediating mechanisms which lead to the activation of the CD.

The diffusion-limited recruitment of a constitutively active domain is supported by several studies about the isolated CD of Ci-VSP that shows catalytic activity against water-soluble substrates without the need of any membrane binding event [4], [12]. However, other results pointed out that the isolated CD completely lacks its dephosphorylation activity against water-soluble substrates in case the linker sequence M240–Y255 is deleted [12]. This suggests an active role for the linker in the VSD-PD coupling, rather than just acting as recruiting element of a constitutively active CD to the membrane. Quite recently, a special role of the linker position K252 regarding the VSD-CD interaction has been proposed [13]. Functional analysis of K252 mutants indicated that this position might interact with the CD instead of binding to the membrane. Accordingly, Liu et al. confirmed these observations with results of crystal structures of the isolated CD [14]. These authors suggested interactions between linker positions K252 and R253 with D400 in the CD. Interestingly, D400 is located in one of the three loops forming the active site of the protein, named TI loop (Fig. 1B, C) [6], [15]. However, the results obtained by the crystallographic structures are very limited, because of the poor resolution of the very flexible linker structure leading to ambiguous interpretations. Therefore, several questions still remain unanswered. If direct interactions really exist between linker and CD, including residue K252 and R253, which role do they play in coupling?

To close this gap of information, we performed atomistic molecular dynamics (MD) simulations on a homology model of the Ci-VSP and three TI loop mutants embedded in a lipid bilayer containing PI(4,5)P_2_ molecules (Fig. S1A). These MD-simulations were complemented with a mutagenesis approach, in which several TI loop mutants (Fig. 1C) were functionally analyzed by electrophysiological measurements on *Xenopus* oocytes. With this combined strategy we are now able to elucidate the dynamics of the VSD-PD interaction in more detail, which would have been difficult to access with classical mutagenesis studies and incomplete crystallographic structures alone. Taken together, our results expand the previous knowledge about molecular mechanisms that enable the inter-domain coupling within the Ci-VSP protein including not only the CD but also the VSD and the linker region.

## Results

### MD Simulations

In this study, classical MD simulations were used to investigate whether the VSD-CD linker of the Ci-VSP protein interacts with putative binding partners in its immediate environment. For this purpose, the number of contacts is defined as all atoms within a 3.5 Å sphere of the corresponding interaction partners. This definition allows the characterization of relevant interactions according to their strength and stability during the MD simulations. While the strength is directly related to the number of contacts (Table 1), the stability is evaluated by their populations and maximal lifetimes (Table 2). The population is discriminating between bound (number of contacts >0) and unbound (number of contacts = 0) states and the maximal lifetime gives information about the maximal length of a contact during the 50 ns of the entire MD simulation.

**Table 1:** Mutations in the TI-loop affect the number of contacts with the linker during the MD simulation.

Model	#contacts	#contacts	#contacts	#contacts	#contacts	#contacts
	**linker–TI**	**N-term–TI**	**C-term–TI**	**K252–TI**	**R253–TI**	**Y255–TI**
WT	37.2±7.2	0.2±0.8	37.1±7.0	4.7±5.2	29.4±4.5	5.7±3.1
NEUT	14.5±6.4	–	14.5±6.4	–	–	14.4±6.3
ALA	34.1±11.9	–	34.1±11.9	2.8±5.8	10.9±5.1	18.7±5.3
D400A	35.6±6.5	0.1±0.8	35.5±6.5	1.5±3.3	26.4±7.0	9.7±5.8

Interactions between the complete linker, its N- and C-terminal parts (240–249 and 250–257, respectively), K252, R253 and Y255 with the TI loop are summarized in terms of average contacts during the last 30 ns of simulation. Contacts are defined as atoms within a sphere of 3.5 Å.

**Table 2:** Relevant calculated populations and maximal contact lifetimes between linker and TI loop residues.

Model	K252–X400	K252–X402	R253–X400	R253–X402	Y255–X400	Y255–F401
States (bound – unbound)						
WT	49–451	202–298	497–3	431–69	0–500	460–40
NEUT	0–500	0–500	0–500	6–494	285–215	460–40
ALA	27–473	81–419	454–46	8–492	497–3	478–22
D400A	4–496	78–422	480–20	488–12	285–215	441–59
Maximal contact lifetime (ns)						
WT	0.6	1.9	24.3	16.6	–	6.9
NEUT	–	–	–	0.2	4.1	13.8
ALA	1.0	4.3	8.9	0.3	24.5	12.4
D400A	0.1	2.0	17.3	21.9	7.7	7.5

Relation of connected and broken states (population) and maximal contact lifetimes for each interaction during the whole simulation of 50 ns are listed. For determining the population, the existence of a contact was checked in 100-ps-steps. Thus, the total number of possible states is 500. For the contact definition, a cut-off sphere of 3.5 Å is used. Beyond this value, a contact is defined as broken.

#### Interaction linker – membrane

The MD simulations performed on all model systems predict a strong interaction between the linker and the PI(4,5)P_2_ molecules at the membrane surface, which is reflected by the high number of contacts between the interacting partners (Table S1). The main contacts are established at an early stage of the simulations and remain stable throughout.

For the wild type (WT) and the TI loop mutants modeled in this work (Fig. 1C), the linker-membrane interaction is predicted to be of electrostatic nature and primarily stabilized by salt bridges (Fig. S1B, C). In particular, positively charged residues of the linker, namely R245, R246, R254, R256 and K257, are identified as main interaction partners for the PI(4,5)P_2_ head groups. While R245 and R246 are located in the N-terminal part of the linker, the other residues (R254, R256 and K257) reside in the C-terminal section. The fact that contacts to PI(4,5)P_2_ molecules are predicted for N- and C- terminal linker residues (Table S1) highlights the importance of the complete linker structure for its interaction with the plasma membrane.

Among the cationic residues of the linker, only K241, K252 and R253 do not establish stable contacts in the form of salt bridges with the PI(4,5)P_2_ head groups. Only in the NEUT model, R253 forms a stable contact to the membrane, which increases the number of membrane interactions for the linker of this mutant (Table S1).

Besides the positively charged residues, uncharged linker positions (*inter alia* M240, A242, S243, S244, S249 and Q250) contribute to the interaction with the membrane either through van der Waals contacts or via hydrophobic interactions. However, these interactions show increased fluctuations and a reduced stability.

#### Interaction linker – CD

Besides the interaction with the membrane, the interplay of the linker with the enzymatic domain, particularly with the TI loop in the CD, is a main objective of this work.

**Figure 2:**
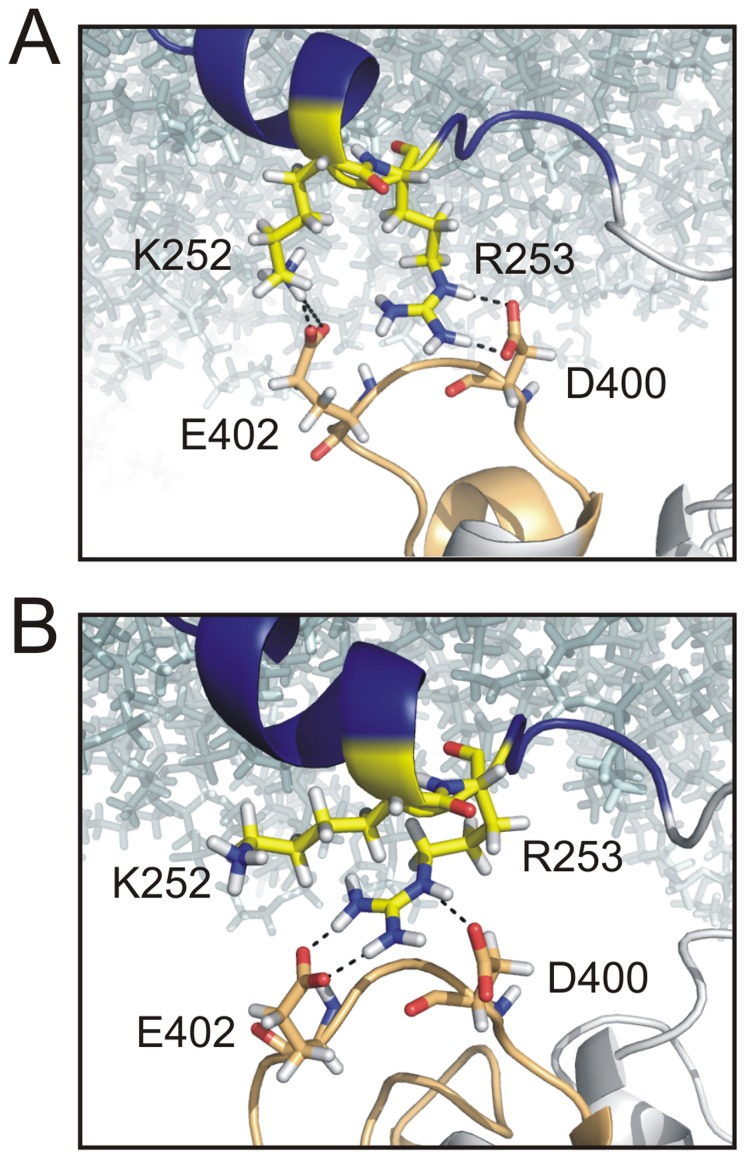
Flipping mechanism of R253 between D400 and E402 in the WT model. Interaction interface between the linker (blue backbone) and the TI loop (beige backbone) in the WT model. Membrane lipids are represented as cyan-colored sticks. A typical snapshot of the most frequently occurring state is shown in *(* ***A*** *)*. Here, salt bridges between R253 and D400 strongly stabilize the contact between linker and TI loop. In this state, K252 is able to interact with E402. *(* ***B*** *)* The stable configuration between R253 and D400 breaks several times during the 50 ns long MD simulation whereby R253 gets in contact with E402. In this constellation of salt bridges, the interaction between K252 and the TI loop is interrupted.

**Figure 3:**
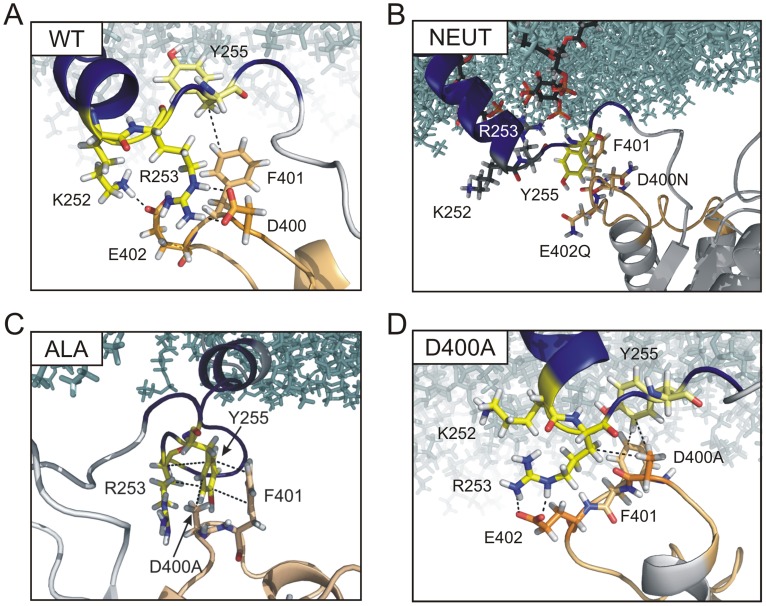
Models of WT and mutants reveal differences in the interface between linker and TI loop. Representative snapshots showing the linker-TI loop interface of *(* ***A*** *)* the WT, *(* ***B*** *)* NEUT, *(* ***C*** *)* ALA, and *(* ***D*** *)* D400A model. For clarity, only K252, R253, Y255, D400X, E402X (X denotes the inserted mutation residue, Fig. 1C) and F401 are shown as indicated in ball-and-stick representation. Furthermore, the backbones of the linker and the TI loop are represented as blue and beige cartoon, respectively. For a better orientation, the membrane lipids are shown as cyan sticks. For the NEUT model, PI(4,5)P_2_ molecules interacting with R253 are indicated, with C atoms in black.

In the WT model, a strong and stable connection between these two regions is observed. This interaction is reflected by the relatively high number of contacts (Table 1). Here, the C-terminal part of the linker plays the most essential role, since it participates in almost 100% of the contacts to the CD (37.2 average contacts against 0.2 coming from the N-terminal linker part). These contacts are mainly characterized by two salt bridges formed between K252, R253 in the linker, and E402, D400 in the TI loop (Fig. 2). Additionally, hydrophobic contacts between Y255 and F401 play a minor, but not negligible role (Table 1, Fig. 3A).

The most stable contact is predicted between R253 and D400 (Fig. 2A), which has a maximal lifetime of 24.3 ns (Table 2). This configuration is further stabilized by the favorable electrostatics between K252 and E402 (Fig. 2A). Occasionally, however, the salt bridge breaks and the guanidino group of R253 flips toward E402, pushing K252 into the solvent (Fig. 2B). In this configuration, R253 weakly interacts with D400 through a hydrogen bond (H-bond) involving the N_ε_ of the arginine. So the interactions R253-D400 and R253-E402 are not mutually exclusive. After a limited life span in this state (with a maximal lifetime of 16.6 ns, Table 2), R253 flips back to D400 due to the electrostatic repulsion by K252, which enables the re-establishment of the salt bridges R253-D400 and K252-E402. The salt bridges described for the WT are interrupted in the NEUT model (Fig. 3B). The lack of appropriate interaction partners in the TI loop for the cationic residues K252 and R253 strongly weakens the interaction between the linker and CD. As shown in Fig. 3B, these two residues either move into the bulk solution or interact with the membrane surface. The decreased coupling between linker and TI loop is primarily characterized by hydrophobic contacts between Y255 and F401 with a maximal lifetime of 13.8 ns, which is shorter than the R253-D400 salt bridge in the WT (Table 2).

Interestingly, in contrast to the NEUT structure, the ALA model displays a rather strong interaction between linker and TI loop, which is reflected by the large number of contacts being in fact comparable to those predicted for the WT model (Table 1). This observation is the result of strong and stable hydrophobic and π-stacking interactions, which involves, *inter alia*, Y255 and the hydrophobic part of R253 in the linker, and D400A, F401 and E402A in the TI loop (Fig. 3C, Table 2). Thus, hydrophobic contacts compensate for the electrostatic interactions which are predominant in the WT protein. Moreover, the important role of electrostatics is nearly abrogated in this mutant, as reflected by the low average number of contacts involving residues K252 and R253 with the TI loop (2.8±5.8 and 10.9±5.1, respectively, Table 1) that are based mainly on hydrophobic interactions (Fig. 3C). Furthermore, the maximal interaction lifetimes of these contacts are drastically reduced in comparison to the WT model (Table 2). This finding is striking considering the fact that the properties of the TI loop in the ALA mutant should be more disruptively changed than in the NEUT model.

In the D400A variant, the interaction between linker and CD can be described as a combination of elements from the WT and the ALA model. Thus, the most stable interaction is characterized by hydrophobic contacts involving Y255, D400A and F401, as observed in the ALA mutant and further by a salt bridge involving R253 and E402, as found in the WT model (Fig. 3D). However, while in the WT the maximal contact lifetime is 24.3 ns for the salt bridge between R253 and D400, this interaction is shortened to 17.3 ns for the D400A mutant and mainly of hydrophobic nature (Table 2, Fig. 3D). This is not surprising, since the salt bridge formation is precluded by the mutated residue. In return, the contact lifetime of the salt bridge between R253 and E402 is increased compared to the WT (21.9 ns vs. 16.6 ns, respectively). These changes in interactions decrease the chance of K252 to build up salt bridges with E402 in the D400A model, because E402 is mostly occupied by interactions with R253, so that the postulated flipping mechanism is blocked (Fig. 3D).

Due to the increased hydrophobicity in the D400A compared to the WT model, stronger hydrophobic contacts could be assumed which may compensate for the changes in interactions, as it has been observed in the ALA model. However, also the hydrophobic connections between linker and TI loop are diminished in comparison to the ALA model (Table 1) and the lifetime of these contacts is strongly shortened to less than 8 ns, which is an indicator of the increased fluctuation at the linker-TI loop interface (Table 2).

#### Structural integrity of the linker

The structural integrity of the linker regarding its initial α-helical conformation is differently affected in the four models, due to the variability in electrostatic conditions of the adjacent TI loop.

In the WT model, the α-helix ranging from H237 to R253 is nearly maintained. The only structural deviation occurs at R245, where the helix is bent in agreement with recent predictions (Fig. S1C) [13].

In the NEUT model, the helix remains straight at R245 and stays nearly unchanged with respect to its initial geometry, which is reflected by the low root-mean-square deviation (rmsd) of only 2.1 Å (Table S2). While the helical structure of the linker in the single mutant D400A is mostly conserved and comparable to the WT, it is completely distorted in the ALA model, where the helix is totally disrupted after position R245.

These observations are reflected by the corresponding rmsd between the backbone atoms of the linker after 50 ns (Table S2). Taking the WT geometry as a reference, the D400A model exhibits an rmsd of 2.6 Å. The other models, namely NEUT and ALA, show much larger deviations of 3.2 and 3.5 Å, respectively. Compared to the initial conformation, the rmsd lies between 2.1 and 3.2 Å for all models, which indicates slight, but noticeable rearrangements (Table S2). These variations in the linker dynamics are ascribed to the different interactions with the TI loop, which additionally leads to diverging membrane-linker coupling.

### Experimental Results

#### Phosphatase activities of TI loop mutants

With the results of the MD simulations at hand, we carried out site-directed mutagenesis of the TI loop positions D400, E402, and D405, which could be candidates for the formation of salt bridges with the linker positions K252, and R253. The functional activity of the mutants was analyzed by co-expressing them with the PI(4,5)P_2_-sensitive potassium channels KCNQ2/KCNQ3 in *Xenopus* oocytes. This system has been frequently described to be optimal for measuring the catalytic activity of Ci-VSP [4], [11], [13].

**Figure 4:**
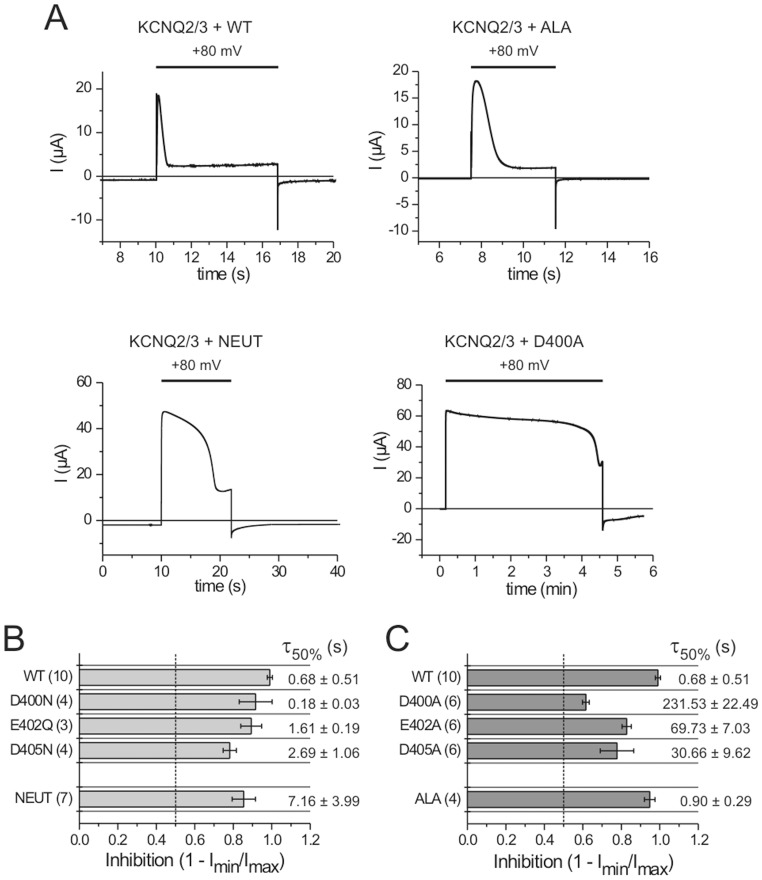
Phosphatase activities of TI loop mutants. *(* ***A*** *)* Representative currents traces of Ci-VSP WT and denoted mutants that were co-expressed with KCNQ2/KCNQ3 potassium channels in *Xenopus* oocytes. Channel currents were recorded in response to a depolarization pulse from a holding potential of −80 to+80 mV for the indicated time interval. *(* ***B*** **–** ***C*** *)* From the resulting current traces, maximal and minimal current amplitudes (I_max_ and I_min_) were determined during the depolarization phase at+80 mV. Inhibition ratios calculated with the values of I_min_ and I_max_ are plotted with the corresponding time durations required for each mutant to inhibit the channel currents to 50% (τ_50%_). Inhibition ratio of 0.5 (corresponding to 50%) is highlighted as dashed line.

By conservative neutralization of individual negatively charged residues in the TI loop (D400N, E402Q, and D405N, Fig. 1C) the CD activities were only slightly reduced showing inhibition efficiencies of ca. 80–90% in comparison to 99% for the WT (Fig. 4B). Furthermore, these single mutants required similar stimulating periods as the WT to inhibit the channel currents to 50% (Fig. 4B).

The simultaneous neutralization of the three positions in the NEUT mutant decreases the CD activity significantly, which shows up in a ca. 10-fold longer stimulation interval the mutant requires to inhibit the KCNQ2/KCNQ3-currents compared to the WT (Fig. 4A, B).

In contrast, single alanine substitutions (D400A, E402A, and D405A) impair the CD activity more dramatically than the NEUT mutation (Fig. 4C). Remarkably, the substitution D400A causes the most destructive effect on the CD activity. This is reflected by the extensive increase of stimulation duration the mutant requires to inhibit the channel currents to 50%, and, moreover, by its lowest inhibition efficiency of ca. 60% compared to all other mutants studied in this work (Fig. 4A, C).

However, none of the single alanine mutations blocked the CD activity completely in a similar way as observed earlier for several linker mutants, e.g. K252C and Y255C [13]. Therefore, we generated the triple mutant ALA which contains D400A, E402A, and D405A simultaneously. Since these mutations were expected to change the TI loop structure and hydrophilicity drastically, we suspected that they would abolish the interaction with the linker completely and, as a consequence, also the catalytic activity.

Surprisingly, the CD activity of the ALA-mutant is more similar to the WT than to all mutants investigated in this work (Fig. 4A, C). Thus, it inhibited the KCNQ/KCNQ3-currents with efficiency of ∼90% and in a similar stimulation interval as the WT (Fig. 4C). Based on these functional data together with the simulation results, we propose for the ALA-mutant that the increased hydrophobicity of the TI loop enables similarly favorable conditions for the interaction with the linker as in the WT, which fully compensate for the lacking salt bridge interactions and, in turn, preserve the catalytic activity of the protein.

#### VSD off-kinetics of TI loop mutants

We further studied the effect of mutations on the VSD kinetics by measuring the “off”-sensing currents of Ci-VSP, since it has been proposed that these signals reflect the strength of binding of the CD to the membrane which occurs during a test potential phase [11], [13]. This interpretation is based on the assumption that the membrane binding of the CD has to be overcome by the VSD during its off-motion, which in return leads to a slow-down in the VSD off-kinetics [11], [13].

**Figure 5:**
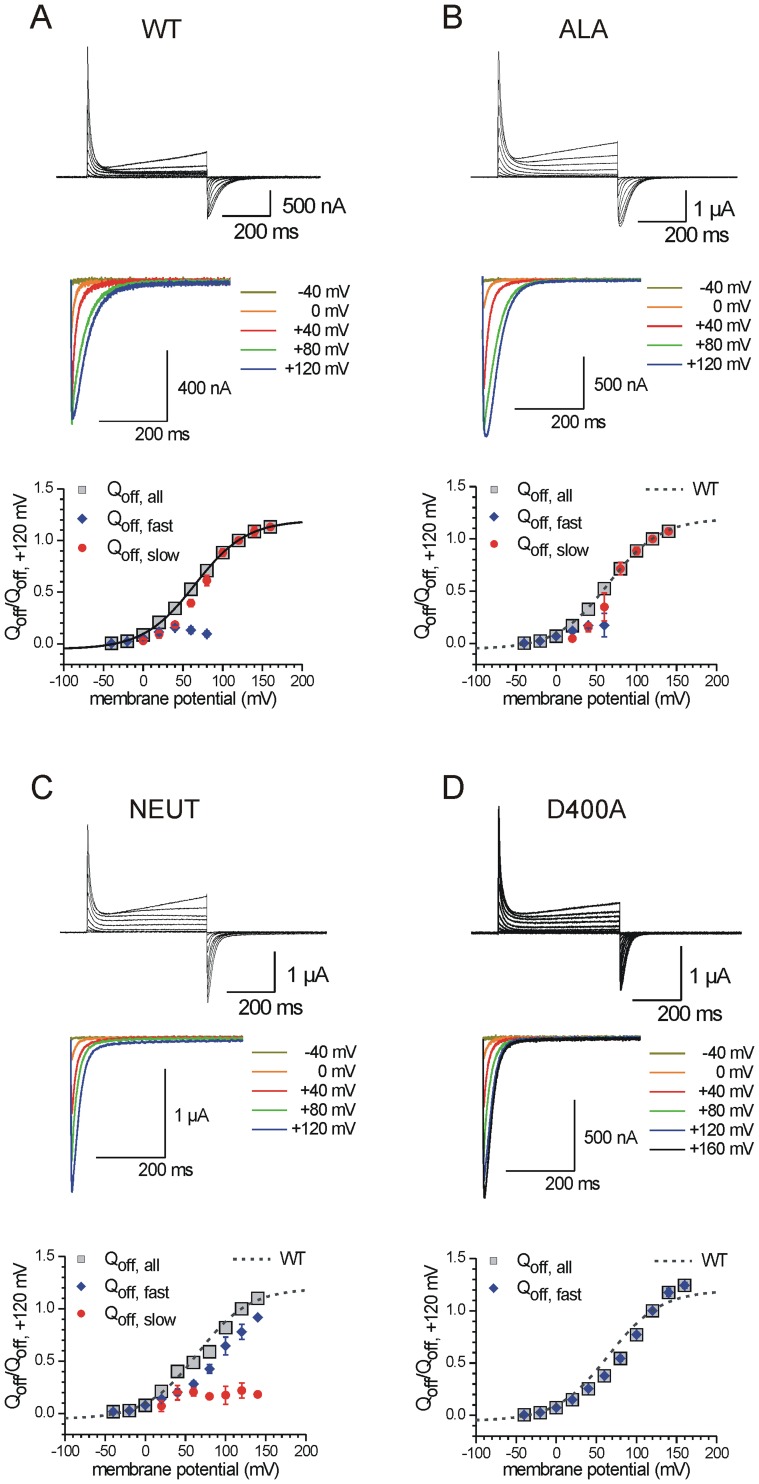
TI loop mutations differently affect the voltage sensor dynamics. On-sensing currents of Ci-VSP were recorded from a holding potential of −60 mV in response to variable test pulses between −40 to+160 mV at maximum (increment: 20 mV, duration: 500 ms). Off-sensing currents were monitored by stepping back to −50 mV after the test potential phase (off-pulse duration: 500 ms). *(* ***A–D*** *)* Representative full current traces are shown for the WT and the denoted mutants. From these signals, selected off-currents are zoomed out (40 mV increments). As described in Materials and Methods, Q_off,all_-V-distributions were calculated by integrating the off-sensing currents. These distributions are plotted against the potential of the preceding test pulse phase. From the whole amount of translocated sensing charges (Q_off,all_), the fast (Q_off,fast_) and the slow (Q_off,slow_) component was determined as described in Materials and Methods. All calculated Q_off_-values were normalized to the Q_off,all_-value corresponding to the test potential of+120 mV. Q_off,all_-V-distributions were approximated with a Boltzmann-type function (see Materials and Methods). Parameters of voltage-dependence (V_0.5_, z_q_) are given in Table S3.

As described earlier [13], the translocation of off-sensing charges in the WT can be described either by mono- or biexponential functions depending on the preceding potential pulse. Therefore, the respective amount of off-sensing charges (Q_off,all_) also consists of a fast (Q_off,fast_) and a slow (Q_off,slow_) component. Whereas Q_off,fast_ is observable after potentials steps between −40 and+80 mV (Fig. 5A), Q_off,slow_ appears after potential pulses from 0 to at least+160 mV. Remarkably, Q_off,slow_ dominates the WT off-kinetics the more positive the preceding potential pulse was (Fig. 5A).

In a previous study, we were able to show that the VSD off-currents decay fast when the CD is deleted [13]. Therefore, we concluded that the slow component in the off-kinetics is caused by processes that are related to the cytosolic part of the protein and, in particular, by its dissociation from the membrane.

Since the occurrence of the Q_off,slow_-fraction at voltages above 0 mV correlates with the appearance of the enzymatic activity in the WT protein [13], we initially assumed that the slow-down in the VSD off-motion is an indicator for having the CD in an active conformation. Interestingly, the kinetic data of the inactive linker mutant K252C contradicted this assumption, since this mutant showed VSD off-kinetics similar to the WT, with a dominating Q_off,slow_-fraction at positive potentials [13]. Thus, a dominating slow fraction in the VSD off-motion is obviously not sufficient to conclude that the CD is in an active conformation.

However, a dominating fast component in the VSD off-kinetics seems to be consistently correlated with a reduction in the enzymatic activity [11], [13]. Therefore, we assume that a proper coupling between VSD and CD is impaired in mutants which show fast VSD off-currents after potential pulses above 0 mV.

In the following, we analyzed the VSD off-dynamics of the TI loop mutants for this correlation. For the single neutralization mutants E402Q and D405N, slower VSD off-kinetics as for the WT are observable, which are mirrored by the moderate increase of the slow time constants (τ_off,slow_) in the off-currents (Fig. S2). Additionally, both mutants have slightly larger slope factors (z_q_) in the voltage-dependence of translocated sensing charge (Q_off,all_-V-distribution, Table S3), which reflects an earlier saturation of the VSD off-motion at less positive potentials than in the WT (Fig. S3). All these results imply a marginally enhanced binding of the CD at the membrane in comparison to the WT.

In case of D400N, the off-kinetics is speeded up at potentials above+60 mV, which is reflected by faster time constants compared to the WT (Fig. S2). In addition, the midpoint potential of the Q_off,all_-V-distribution (V_0.5_) is remarkably positively shifted by ca.+15 mV, although the z_q_-value is in a similar range as for the WT (Table S3). The kinetic data of D400N corresponds to the special role of this position in the VSD-CD coupling, because the neutralization of D400 obviously leads to less restricted VSD off-motion.

For the single substitutions E402A and D405A, a similar behavior as for the neutralizing mutations E402Q and D405N is observed. The τ_off,slow_-values are a bit larger as for the WT (Fig. S2) as well as the z_q_-values (Table S3), which also indicates a slight increase in membrane binding of their catalytic domains.

In case of D400A, the VSD off-kinetics is most profoundly affected in comparison to all other mutants studied in this work. The Q_off,all_-V-distribution is significantly positive-shifted by about+30 mV (Fig. 5D, Table S3). Furthermore, the off-currents decay monoexponentially with very fast time constants compared to the WT (Fig. 5D, Fig. S2). These results indicate a disruption of the CD-mediated slow-down in the VSD off-motion for this mutant.

Interestingly, in line with the MD-simulations and CD activities, the Q_off,all_-V-distribution of the ALA-mutant is most similar to the WT compared to all other mutants (Fig. 5B). Thus, their τ_off_-values are nearly identical for both, the fast and the slow time constants (Fig. S2). Additionally, the midpoint potential V_0.5_ of the Q_off,all_-V-distribution is almost the same as for the WT (Table S3). Only the difference in z_q_-values between the WT and ALA (with 0.78 and 0.90, respectively) indicates an earlier onset of saturation of the Q_off,all_-V-distribution for the ALA-mutant, which suggests a slightly stronger binding of the CD in the mutant.

As predicted from the MD simulation, reduced interaction between TI loop and linker in the NEUT-mutant is also supported by the corresponding VSD off-kinetics. Thus, the off-currents are speeded up similar to the D400A mutant (Fig. 5C, D). However, in contrast to D400A, the Q_off,all_-V-distribution contains both, a slow and a fast Q_off_-fraction over the whole potential range (Fig. 5C), which correlates with a reduction in the catalytic activity, but not to the same extent as for D400A (Fig. 4).

As described earlier for several linker mutants, a reduction in their catalytic activity is correlated with a dominant fast Q_off_-component [13]. Here, such a correlation between catalytic activity and VSD off-kinetics is again observed for the NEUT-mutant and, even more pronounced, for D400A.

In summary, the VSD off-kinetics obtained for all TI loop mutants are consistent with the results of their CD activities. Thus, the more dominant the fast component of the off-currents becomes, the more the catalytic activity is reduced. According to the analysis of the CD activity, the VSD off-kinetics of the D400 mutants refers to the special role of this residue in the interaction between the linker and the TI loop, and, therefore, also on the VSD-CD coupling.

## Discussion

The efficiency of the voltage-dependent activity of Ci-VSP depends on the quality of coupling between the VSD and the CD. For these processes, the region that links both modules is assumed to play a crucial role [4], [11]–[13]. Particularly, the positively charged character of the linker suggests that it interacts electrostatically with negatively charged counterparts in its immediate environment. Several studies support the hypothesis that the binding of the linker to the membrane surface is crucial for coupling in Ci-VSP, since it might result in the recruiting of the CD to its substrate [11]–[13]. Besides that, previous results further suggested interactions between the linker positions K252 and R253 with residues inside the CD [13], [14]. However, the knowledge about the dynamic VSD-CD interaction is still limited.

In this study, we gain more insight into this aspect with a combined approach of MD simulations and electrophysiology. We created a three-dimensional homology model of the Ci-VSP structure which includes the VSD and CD, both connected by an α-helical linker as proposed earlier [13]. This was done for the WT structure as well as for the three mutants NEUT, ALA and D400A (Fig. 1C).

In our classical MD simulations, interactions between the linker and the negatively charged membrane surface are observed for all models that confirms suggestions of previous studies [11]–[13]. It should be mentioned that we analyzed in our MD simulation only the interactions of the linker with PI(4,5)P_2_, but not with other PIP-species, such as PI(3,4)P_2_, PI(3,5)P_2_ or PI(3,4,5)P_3_. Thus, we cannot conclude whether the linker residues might exhibit a binding preference for one of these membrane lipids or not. This question must be addressed in forthcoming studies.

As suggested earlier, we observed a bending of the linker structure in the WT. However, the kink did not occur at position S249 as proposed earlier [13], but at position R245. Interestingly, this structural interruption enables stable interactions between the C-terminal linker residues K252, R253 and Y255 with D400, F401 and E402 in the TI loop of the CD. Remarkably, a mixture of electrostatic and hydrophobic interactions seems to be important for the linkage between linker and TI loop. Whereas K252 and R253 in the WT model form salt bridges with E402 and D400, respectively, Y255 contributes with hydrophobic contacts to F401.

These model predictions are supported by the electrophysiological data. Thus, single mutations in the TI loop affect the activity of the CD and the VSD off-kinetics depending on the properties of amino acids used for the replacement. Thereby, single conservatively neutralizing mutations have less impact on the coupling between the protein modules, presumably because they form H-bonds with the linker instead of salt bridges. Contrary, single alanine mutations interfere dramatically with these processes, probably because the formation of H-bonds and salt bridges are disrupted. Moreover, hydrophobic interactions with one introduced hydrophobic residue alone seem to be insufficient to compensate for the lack of electrostatic interactions.

Interestingly, all single alanine mutations in the TI loop lead to a CD activity as it was observed earlier for most of the C-terminal cysteine linker mutants N251C-K257C [13]. These results suggest that TI loop- and C-terminal linker mutations similarly affect the VSD-CD-interaction.

From all positions mutated individually in the TI loop, D400 seems to be most crucial for maintaining the coupling. Thus, both D400Q and D400A cause the most dramatic effect on the VSD-off-kinetics and, especially D400A, also on the CD activity. Finally, the MD simulations provided a clue for understanding the importance of this position. While in the WT model a flipping of R253 between D400 and E402 is observed, this process is inhibited in the D400A model, where R253 preferentially binds to E402. If the stable interaction between R253 and E402 is the reason for the almost complete lack of catalytic activity of the D400A mutant, it seems to be obvious that the connection between R253 and D400 is essential for an efficient coupling between the VSD and the CD. In this way, it is also now possible to rationalize why the mutation K252C abolished the CD activity completely [13]. The presence of K252 seems to be crucial to push R253 towards D400, while forming the salt bridge K252-E402.

This interpretation agrees with results obtained recently from crystal structures of the Ci-VSP CD by Liu et al. [14]. These authors also suggested interactions between K252 and R253 with D400. Controversially, they observed other interaction modes between these three residues for their different crystal structures. Thus, K252 seemed to interact with D400, similar as R253. Furthermore, the competition between K252 and R253 for D400 was described to be stabilized by interactions via H-bonds to the G365 backbone carbonyl. This interaction is not observable in our simulation of the WT model, where the distance between K252 or R253 to the G365 backbone carbonyl was predicted to be 15 Å through the entire simulation.

It is important to note that during crystallization of the M240-I576-fragements of Ci-VSP’s soluble cytosolic domain by Liu et al., the linker motif inevitably lacked the structural stabilization mediated by the VSD and the membrane surface allowing unconstrained movements of this flexible region. In the present study, the influence of the membrane surface on the structural integrity of the linker becomes obvious. In particular, in the MD-simulation of the NEUT model, where the polarity of the neutralized TI loop is no longer able to compensate for the attractive force mediated by the phosphate groups of the PI(4,5)P_2_ molecules, the linker residue R253 is oriented towards the membrane surface rather than to the TI loop, which induces different structural rearrangements in the C-terminal linker compared to the WT. This reorientation seems to cause both, a reduction of catalytic activity and a disrupted coupling of the Ci-VSP modules as mirrored by the acceleration of the VSD off-currents (Fig. 5C). Hence, it can be assumed that the electrostatic attraction by the membrane is crucial for keeping the linker in a configuration that enables stable interactions with the TI loop. Taking these results into account, the interpretation of Liu et al. about flexible linker movements based on rigid crystal structures alone remains elusive.

Another indicator for the complexity of the interaction between the linker and the TI loop is observable in the dynamics of the ALA mutant. Here, due to the lack of salt bridges between K252, R253 with E402A and D400A, the linker structure is also completely reorganized compared to the WT. However, the increased hydrophobicity of the TI loop is able to compensate for the loss of electrostatic attractions by forming stable contacts between Y255, D400A and F401, which are occasionally mediated by π-stacking interactions between the two phenyl rings of Y255 and F401 (Fig. 3C). These contacts are further stabilized by interactions of the hydrophobic parts of the side chains from K252 and R253 with D400A and E402A. Although the C-terminal linker’s α-helical conformation is disrupted, the hydrophobic linkage to the TI loop obviously enables similar VSD-CD-interactions as in the WT, which is mirrored by the maintained CD activity as well as in almost identical VSD off-currents. Concordantly, the importance of the phenyl ring at linker position Y255 for Ci-VSP function was proven quite recently. While the mutation Y255C completely abolished phosphatase activity, the Y255F substitution preserved enzyme function [13].

Here, the results of the NEUT and ALA mutants illustrate the importance of balanced electrostatic and hydrophobic interactions between linker, membrane and CD. This complex interaction pattern is only revealed by considering the dynamics of the molecule and reaches beyond the previous concept of electrostatic repulsion or attraction between different regions of the Ci-VSP protein.

**Figure 6:**
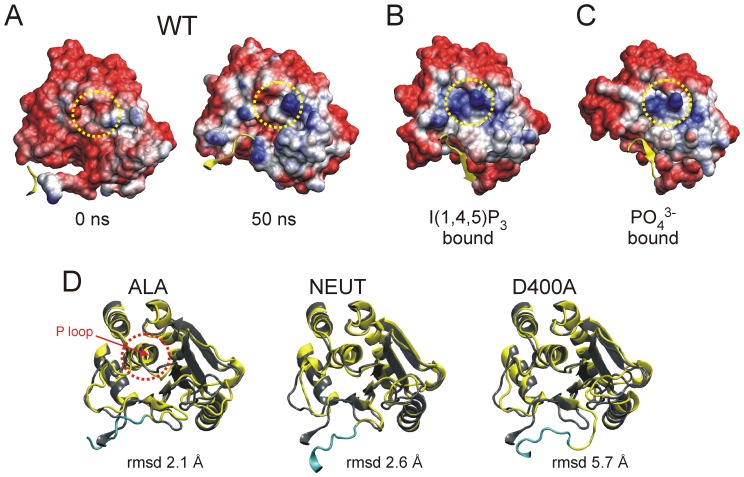
Interaction between linker and TI loop influences the structural integrity of the active site. *(* ***A–C*** *)* Electrostatic potential surfaces of the phosphatase domain calculated with the APBS tool [26] are shown. Representations of *(* ***A*** *)* the wild type model at 0 and after 50 ns of the MD simulation, *(* ***B*** *)* the crystallographic structures by Liu et al. of the IP_3_ bound conformation (PDB 3V0H) and *(* ***C*** *)* the PO_4_^3–^bound state (3V0G) [14]. Negatively and positively charged regions are colored in red and blue, respectively. The yellow element marks the linker residues 252–257. Encircled in yellow is the immediate environment of the catalytic cysteine C363. *(* ***D*** *)* The aligned backbone structures of the phosphatase domain of the WT (grey) and the corresponding TI loop mutant (yellow) are shown. The P loop containing C363 is highlighted with a dashed circle in red. The linker motif K252-K257 of the mutants is indicated in cyan. Additionally, the figure contains the rmsd values for the structural deviation between wild type and the respective mutant.

However, the question remains which processes lead to the activation of the CD after the linker has bound to the membrane. Two models of the activation process have been proposed: (1.) a recruitment of a constitutively active phosphatase to the membrane, and (2.) a ‘gating’ process, in which the CD in addition has to undergo conformational changes to enable catalysis. These processes can be illustrated in more detail by examining the substrate binding pocket of Ci-VSP based on the available crystal structures and our modeling data (Fig. 6, Fig. S4). Our results clearly show a highly negative electrostatic potential of the membrane-facing surface of the phosphatase domain in the initial state (Fig. 6A). Such a pattern can similarly be observed for the crystal structures of the Ci-VSP CD when no substrate molecule is bound to the active site (Fig. S4A) [14], [15]. This raises the question why the highly negatively charged PIP-substrate should get access to the active site under such unfavorable electrostatic conditions.

For the WT and all TI loop mutants studied here, the positively charged residues around the catalytic cysteine C363 emerge from the substrate binding pocket during the MD simulations (Fig. 6A, Fig. S4B–D). The aforementioned results of the NEUT model imply that these structural rearrangements are caused by the electrostatic attraction mediated by the PI(4,5)P_2_-head groups during the recruitment of the CD to the membrane. We presume that the conformations obtained at the end of the MD simulations for the WT and the TI loop mutants represent intermediate open states of the substrate binding pocket, because the active site configuration in the crystal structure with an I(1,4,5)P_3_-molecule bound in the substrate binding pocket [14] suggests a further concentration of positively charged amino acids around the catalytic center (Fig. 6B). Interestingly, the binding pocket seems to adapt to the substrate, since the positive charges are even more concentrated, when a small PO_4_^3–^molecule is bound instead of I(1,4,5)P_3_ (Fig. 6C). This suggests a certain degree of flexibility in the substrate binding pocket.

However, the exposure of cationic residues, which appears as prerequisite for substrate binding, requires that all negatively charged amino acids surrounding the active site in the initial state need to be pushed away from the substrate binding pocket by dynamic interactions with the membrane or with the linker.

In case of the Ci-VSP homolog PTEN, it has already been suggested that the flexible TI loop could aid in adapting the size of the active site to its substrate [6]. Moreover, it was proposed that the reorientation of this loop away from the substrate binding pocket might enable the dephosphorylation of the sterically demanding PIP-head groups [6].

For Ci-VSP, Liu et al. suggested that residue E411 is one of the candidates in the TI loop for switching between an open and a closed state of the Ci-VSP CD since it could compete with the substrate for binding at the active site [14]. In our model simulations, however, E411 does not dramatically change its position on a 50 ns time scale. This could be due to the fact that we here observe only an intermediate open conformation of the active site. To observe all structural rearrangements in the substrate binding pocket, a simulation of the entire docking process might be required. Substrate docking takes place on a large time scale, which is impossible to simulate by our present computational resources.

Nonetheless, our study sheds light on several details regarding the role of the interaction between linker and TI loop for the early conformational changes that need to occur in the substrate binding pocket during the recruitment of the CD to the membrane. Although the electrostatic potential surface for the WT and the TI loop mutants does not differ dramatically at the end of our simulations (Fig. 6A, Fig. S4B–D), a close-up view of the interaction zone between linker and TI loop reveals more clearly the role of both flexible regions for the coupling process. In Fig. 6D the coordination of the backbones of the phosphatase domain is shown, which includes the flexible regions of linker, TI and WPD loop surrounding the active site motif around C363 (named as P-loop). According to the structural deviation of the region that is oriented towards the linker, the ALA model shows the highest similarity with respect to the WT, which is mirrored in the lowest rmsd value of 2.1 Å. For the NEUT and the D400A model, the values increase from 2.6 to 5.7 Å, respectively. This corresponds to the differently decreased phosphatase activities of both mutants. Therefore, the reduced interaction strength between linker and TI loop in the NEUT and, even more so, in the D400A model apparently destabilizes one of the boundaries of the substrate binding pocket, which might explain the impaired catalytic activity of these mutants.

In summary, the multifaceted interplay between linker and TI loop seems to invoke structural rearrangements in the substrate binding pocket, which are crucial to switch on catalytic activity. This, in effect, would reject the hypothesis of a constitutively active phosphatase in Ci-VSP. Since the formation of stable salt bridges and hydrophobic interactions between linker and TI loop is coupled to the activation state of the VSD, we propose that the linker acts as a dynamic scaffold during the voltage-dependent coupling mechanism, and - in effect - serves as a key to unlock the enzymatic activity of the Ci-VSP protein.

## Materials and Methods

### Model Building

To investigate the dynamics of Ci-VSP in the plasma membrane with MD simulations, an initial geometry is required. Because a complete crystallographic structure of Ci-VSP is lacking, a homology model of the protein embedded in the plasma membrane was developed. For this procedure, the programs YASARA (http://www.yasara.org) and VMD 1.8.7 [16] were used. The crystallographic structures of the voltage-dependent Shaker family K^+^channel (PDB entry 2R9R) [17] and the Ci-VSP phosphatase-C2 domain complex (3AWE) [15] served as templates for the sensory and PD-C2 domains, respectively. Since the structural template for the VSD was taken from a K_V_-channel structure representing the activated configuration [17], the VSD-CD linker and the CD should encounter the structural constraints present during the activation process of the enzymatic domain.

The unknown or very poorly resolved linker was modeled as α-helix as predicted earlier [13]. Small gaps in the structural templates, such as the flexible TI loop in the crystal structure of the PD-C2-complex by Matsuda et al. [15], were inserted into the geometry and energy minimized *in vacuo*. Although several crystal structures by Liu et al. contain the TI loop, an alignment of this region derived from the different crystals revealed high structural fluctuations in the loop while the position of the neighboring helices were conserved (Fig. S5A). Comparison of the TI loop in our initial WT model with the different structures resolved by Liu et al. shows that our model fits well into the variety of possible conformations represented in the various crystals (Fig. S5A). Together with the high B-values of the TI loop elements from the structural data by Liu et al. (Fig. S5B), we assume that the loop is flexible and not restricted to a certain conformation. Because of these observations, there is no obvious reason why one of the conformations from the different crystal structures by Liu et al. should be preferred for the modeling. Therefore, we chose to continue with our initial model based upon the PD-C2-complex by Matsuda et al. [15].

The cytosolic N-terminus preceding the VSD (107 amino acids) was omitted in our model due to the lack of an appropriate template structure.

The corresponding double layer membrane consists of 291 neutrally charged POPE (1-palmitoyl-2-oleoyl- sn-glycerol-3-phosphatidylethanolamine) chains and 13 negatively charged PI(4,5)P_2_ (phosphatidylinositol-4,5-bisphosphate) molecules located at the in the cytosolic leaflet. The PI(4,5)P_2_ chains were modeled facing the linker and PD-C2 complex, which are both positively charged at physiological pH. This initial placing of the PI(4,5)P_2_ molecules was adopted to speed up the electrostatic interactions with the positively charged regions of Ci-VSP and to avoid long rearrangement processes within the membrane. The PI(4,5)P_2_ head groups were treated with the partial charges derived by Lupyan et al. [18].

To investigate changes in the dynamics and the interaction between the membrane and Ci-VSP and, furthermore, to resolve more details about the coupling between the linker and the TI loop, we generated, additionally to the wild type (WT), three mutants by selectively replacing the negatively charged amino acids of the TI loop (D400, E402, D405). In the first mutant (NEUT model), we simply neutralized the charges of the loop region simultaneously while keeping the amino acid side chains as similar as possible (D400N, E402Q, D405N, Fig. 1C). In the second model (ALA model), we mutated all indicated residues to alanines (D400A, E402A, D405A), which strongly alters the electrostatic nature of the region. To demonstrate the importance of D400A, we constructed a third modification of Ci-VSP by changing D400 into alanine (D400A model).

In order to approximate experimental conditions, we solvated the Ci-VSP-membrane models in a TIP3P water box [19]. Additionally, we added Na^+^and Cl^−^ ions to obtain a neutrally charged system with an ionic strength of 100 mM mimicking the experimental conditions. In total, each system contains more than 153.000 atoms and has a dimension of ca. 177×101×147 Å^3^.

### Molecular Dynamics Simulations

Atomistic molecular dynamics simulations were performed with NAMD2.7 [20] using the CHARMM27 force field including the CHARMM force field for lipids [21].

In a first step, the energy of the models containing Ci-VSP, the membrane and the solution was energy minimized with the conjugated gradient routine in 30.000 steps. During the minimization, all heavy atoms were constrained with 25 kcal·mol^−1^ Å^−2^ to their positions. Afterwards, the systems were heated up to 300 K in 60 ps, while the position constraints were decreased stepwise from 7.5 to 2.5 kcal·mol^−1^ Å^−2^.

In the following solvent equilibration, the water and ions were allowed to translate and rotate without any restrictions and the constraints on the protein and the membrane were further decreased until all atoms in the systems were allowed to move freely in the cell. After this preparation, an unconstrained production MD run of 50 ns was carried out to investigate the dynamics of the system. The simulations were run on 128 CPUs (16 nodes with 8 tasks) in parallel and required in total more than 6.400 CPU hours.

To obtain a realistic behavior of the membrane, periodic boundary conditions in all dimensions were applied keeping the cell size in the membrane directions constant. This cell setup was realized by an NPaT ensemble, where besides the surface area the numbers of particles (N), the pressure (P) and the temperature (T) were kept constant during the simulation. Langevin piston dynamics [22] enabled these conditions. Furthermore, an integration time step of 2 fs was employed, which was enabled by the SHAKE algorithm treating all bonds including hydrogen atoms as rigid [23].

Short-range electrostatics and van der Waals interactions were simulated with a cut-off of 12 Å, while long-range electrostatics was calculated with the particle mesh Ewald summation [24].

### Mutagenesis

Ci-VSP cDNA was subcloned into the plasmid vector pFROG3, which has been optimized for heterologous protein expression in *Xenopus* oocytes [25]. All mutations were generated using the QuikChange site directed mutagenesis kit (Stratagene, La Jolla, CA), and verified by sequencing (Eurofins MWG Operon, Germany).

### cRNA Synthesis and Injection of *Xenopus* Oocytes

Ci-VSP cDNA was linearized with *Ksp*AI (Fermentas). Afterwards, cRNA was synthesized with the T7 mMessage mMachine kit (Ambion, USA). KCNQ2 and KCNQ3 cDNA (subcloned into the plasmid vector pTLN) were linearized with *Not*I (Fermentas) and transcribed into cRNA with the SP6 mMessage mMachine kit (Ambion). The preparation of *Xenopus* oocytes was done as described in detail recently [13].

For measuring transient sensing currents, 50 nL of 0.5 µg/µL Ci-VSP cRNA were injected per cell; for CD-activity measurements, 50 nL of a KCNQ2:KCNQ3:Ci-VSP cRNA mixture (0.05∶0.05∶0.5 µg/µL) were injected. After injection, oocytes were stored for 3–4 days at 18°C in ORI buffer containing 50 mg/L gentamycin, as described previously [13].

### Electrophysiology

3–4 days after injection, currents were recorded at 21–23°C with the two-electrode voltage-clamp technique using a Turbotec 10CX amplifier (NPI instruments, Tamm, Germany). The experimental solutions and procedures for analyzing VSD-kinetics (from Ci-VSP’s “off” sensing currents) and CD-activities (from the inhibition of KCNQ2/KCNQ3 currents) used in this study were recently described in detail [13]. Measuring protocols are denoted at the respective positions in the results section.

### Data Acquisition and Analysis

Currents were acquired using the pClamp 10 software (Axon Instruments, USA). Data were analyzed with pClampfit 10 (Axon Instruments), Excel (Microsoft, USA) and Origin 7.0 (Microcal, USA). As described previously [13], the time constants τ_off_ for the VSD off-currents were determined by exponential approximation:
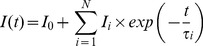
(1)with N = 1 for monoexponential and N = 2 for biexponential kinetics.

The decisive criteria for using a mono- or biexponential approximation was the χ^2^-value determined with a χ^2^-minimization of the respective data set, without applying a weight to individual experimental data points. The parameters of the function that gave the minimal χ^2^-value during the fitting session were used subsequently, because this function described the VSD off-kinetics most precisely.

According to Eq. 1, the data set used for the fitting session started from t = 5 ms to calculate the initial current amplitudes at the onset of the off-pulse directly from the fitted function, and to eliminate residual capacitive artefacts. The charge translocated during the VSD off-motion, Q_off,all_, was determined from the fitting parameters from Eq. 1 with:
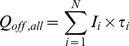
(2)Q_off,fast_ was calculated from the product of τ_off,fast_ and its corresponding initial current amplitude I_off,fast_. Q_off,slow_ was determined analogously with the values of τ_off,slow_ and I_off,slow_.

The values which describe the voltage-dependence of the VSD off-kinetics, V_0.5_ (midpoint potential) and z_q_ (slope factor), were determined by fitting the Q_off,all_-V-relationship with a Boltzmann-type function:
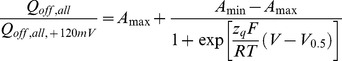
with the applied test potential V, the Faraday constant F, the gas constant R, the absolute temperature T in K, and A_min_, A_max_ as the minimal and maximal values the function adopts during approximation.

For statistics, at least three independent measurements were analyzed. Oocytes were obtained from at least two different cell batches. Unless mentioned differently, means ± standard deviations are presented.

## Supporting Information

Figure S1 **Three-dimensional Ci-VSP model with the VSD embedded in a lipid bilayer and with interactions between linker residues and PI(4,5)P_2_ molecules.** ***(A)*** Structural model of the protein backbone for the wild type before (0 ns) and after (50 ns) the MD-simulation. The VSD (colored in dark gray) is embedded in the lipid bilayer containing 291 neutrally charged POPE chains (cyan) and 13 negatively charged PI(4,5)P_2_ molecules (yellow). The CD is coupled to the VSD via the linker motif M240–K257 (blue). PD and C2 domain are marked in medium and light gray, respectively. *(B, C)* Structural geometries between single linker residues (yellow, and N251 in orange) and PI(4,5)P_2_ molecules at the inner membrane surface *(B)* before and *(C)* after the MD-simulation. Heavy atoms are marked in the following colors: nitrogen, blue; oxygen, red; phosphorus, orange; hydrogen, white. (The structure coordinates of our wild type model will be available upon request.)
(TIF)

Figure S2 **Differences in voltage-dependent time constants of the VSD off-motion for TI loop mutants.** Transient off-currents of Ci-VSP were approximated with mono- or biexponential functions as described earlier [13]. Fast and slow time constants (τ_off_) for the VSD-off-kinetics were determined depending on the membrane potential applied during the test pulse phase. Averaged τ_off_-values are given as filled symbols. For comparison, the corresponding voltage-dependent time constants of the WT are shown as dotted lines.
(TIF)

Figure S3 **Voltage-dependence of translocated off-sensing charges for TI loop mutants.** Sensing charges translocated during the off-motion of the VSD were calculated as described earlier [13]. The averaged fast (Q_off,fast_ in blue) and slow fraction (Q_off,slow_ in red) of the off-sensing charge are plotted against the membrane potential as well as the sum of both (Q_off,all_ in gray). The individual Q_off_-values, which were obtained per oocyte, were normalized to the respective Q_off,all_-value at+120 mV. Q_off,all_ was approximated with a Boltzmann-type function as described in Materials and Methods. Fitting parameters V_0.5_ and z_q_ are given in Table S3.
(TIF)

Figure S4 **Electrostatic potential surfaces for the catalytic domain of Ci-VSP in the substrate-unbound conformation and the modeled TI loop mutants.** Electrostatic potential surfaces for the phosphatase domain of Ci-VSP were calculated with the APBS tool [26]. Representations of *(* ***A*** *)* the unbound conformations based on the crystal structures by Matsuda et al., 2011 [15] (left panel) and Liu et al., 2012 [14] (right panel; PDB entries are given, respectively) as well as *(* ***B–D*** *)* for the denoted TI loop mutants before (0 ns) and after (50 ns) of MD simulation. It should be noted that the differences in electrostatic potentials between the WT (Fig. 6A) and the mutants at 0 ns is due to the reduction of the negatively charged character in the TI loop caused by the respective mutation.
(TIF)

Figure S5 **Structural alignment between the TI loop region of the initial Ci-VSP WT model and crystallographic structures.** ***(A)*** The TI loop of our initial Ci-VSP WT model (red) is structurally aligned with the respective region from the crystallographic structures by Liu et al. [14] (cyan). To obtain a suitable alignment, the neighboring α-helices were superimposed, with helix a and b containing the residues T386–T399 and T412–Y429, respectively. ***(B)*** The B-values of the C_α_-atoms for all residues resolved in the crystallographic structures by Liu et al. [14] are plotted. The red box marks the strongly fluctuating region of the TI loop.
(TIF)

Table S1 **Averaged number of contacts between linker and PI(4,5)P_2_ residues.** Number of contacts (#contacts) between the linker and PI(4,5)P_2_ residues averaged over the last 30 ns. Additionally, the interaction of the N-terminal (240–249) and the C-terminal (250–257) parts of the linker and PI(4,5)P_2_ residues is shown. As in Table 1, contacts are defined as atoms within a sphere of 3.5 Å.
(DOC)

Table S2 **Structural changes of the linker in terms of the root-mean-square deviation.** The differences between the end geometries in comparison to the WT enzyme after 50 ns are shown. Additionally, the over the last 30 ns averaged rmsd to the initial conformation is displayed. All rmsd values refer to the backbone atoms of the linker region (240 to 257).
(DOC)

Table S3 **Boltzmann-parameters for the voltage-dependent translocation of sensing charges in Ci-VSP.** Amount of voltage-dependent sensing charges (Q_off,all_-V-values) were determined from the transient off-currents of Ci-VSP as described in detail earlier [13]. The resulting Q_off,all_-V-distributions were approximated with a Boltzmann-type function (see Materials and Methods) to determine the parameters V_0.5_ (midpoint potential) and z_q_ (slope factor) which describe the voltage-dependence of the off-currents (n: numbers of independently performed measurements).
(DOC)
