# Supplementary figures and images for: The Linker Pivot in Ci-VSP: The Key to Unlock Catalysis
Source: PLoS One. 2013 Jul 29;8(7):e70272. doi: 10.1371/journal.pone.0070272 (PMC3726396; doi:10.1371/journal.pone.0070272)

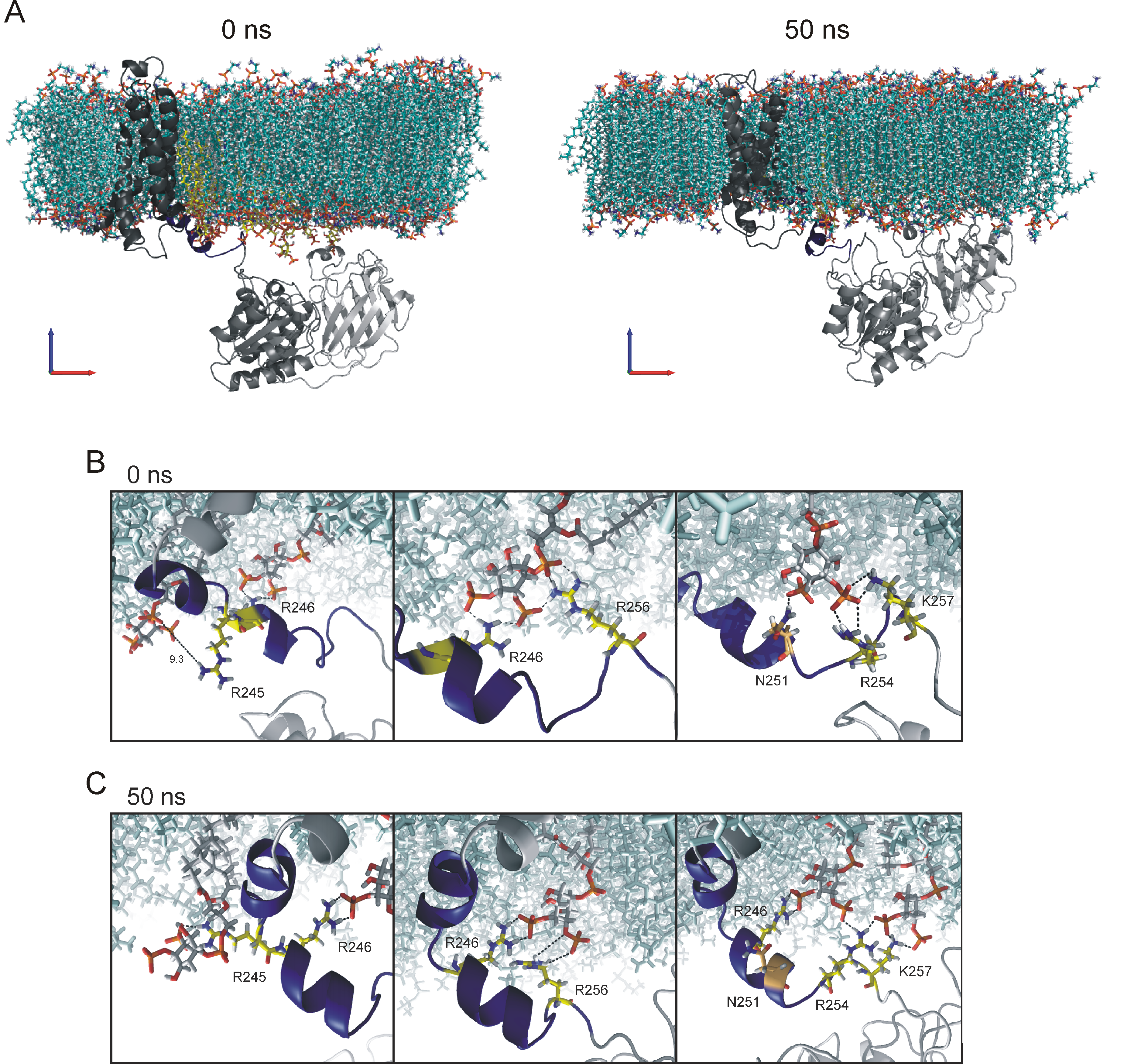

Supplement: Figure S1 — Three-dimensional Ci-VSP model with the VSD embedded in a lipid bilayer and with interactions between linker residues and PI(4,5)P2 molecules. (A) Structural model of the protein backbone for the wild type before (0 ns) and after (50 ns) the MD-simulation. The VSD (colored in dark gray) is embedded in the lipid bilayer containing 291 neutrally charged POPE chains (cyan) and 13 negatively charged PI(4,5)P2 molecules (yellow). The CD is coupled to the VSD via the linker motif M240–K257 (blue). PD and C2 domain are marked in medium and light gray, respectively. (B, C) Structural geometries between single linker residues (yellow, and N251 in orange) and PI(4,5)P2 molecules at the inner membrane surface (B) before and (C) after the MD-simulation. Heavy atoms are marked in the following colors: nitrogen, blue; oxygen, red; phosphorus, orange; hydrogen, white. (The structure coordinates of our wild type model will be available upon request.) (TIF) [file pone.0070272.s001.tif]

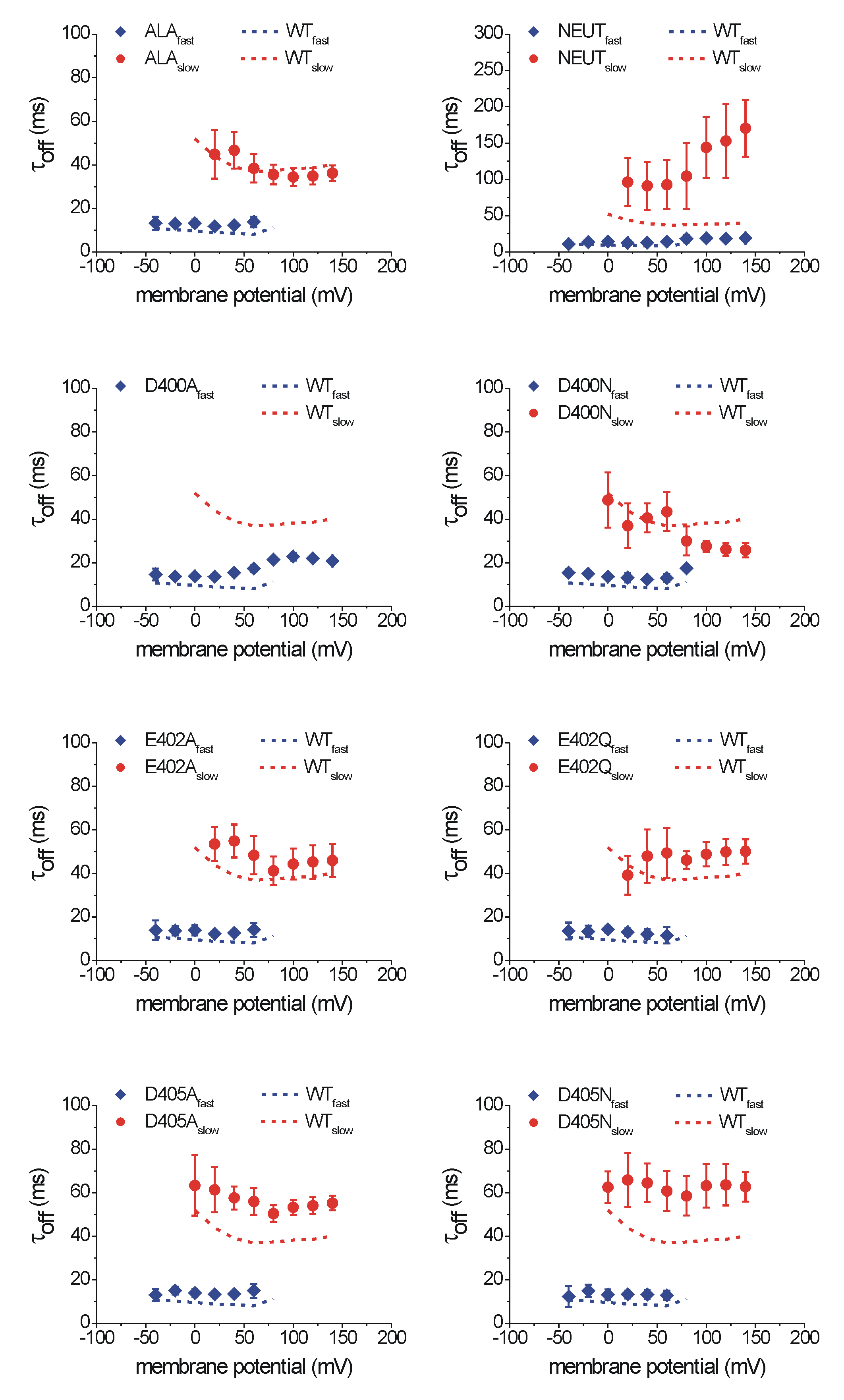

Supplement: Figure S2 — Differences in voltage-dependent time constants of the VSD off-motion for TI loop mutants. Transient off-currents of Ci-VSP were approximated with mono- or biexponential functions as described earlier [13]. Fast and slow time constants (τoff) for the VSD-off-kinetics were determined depending on the membrane potential applied during the test pulse phase. Averaged τoff-values are given as filled symbols. For comparison, the corresponding voltage-dependent time constants of the WT are shown as dotted lines. (TIF) [file pone.0070272.s002.tif]

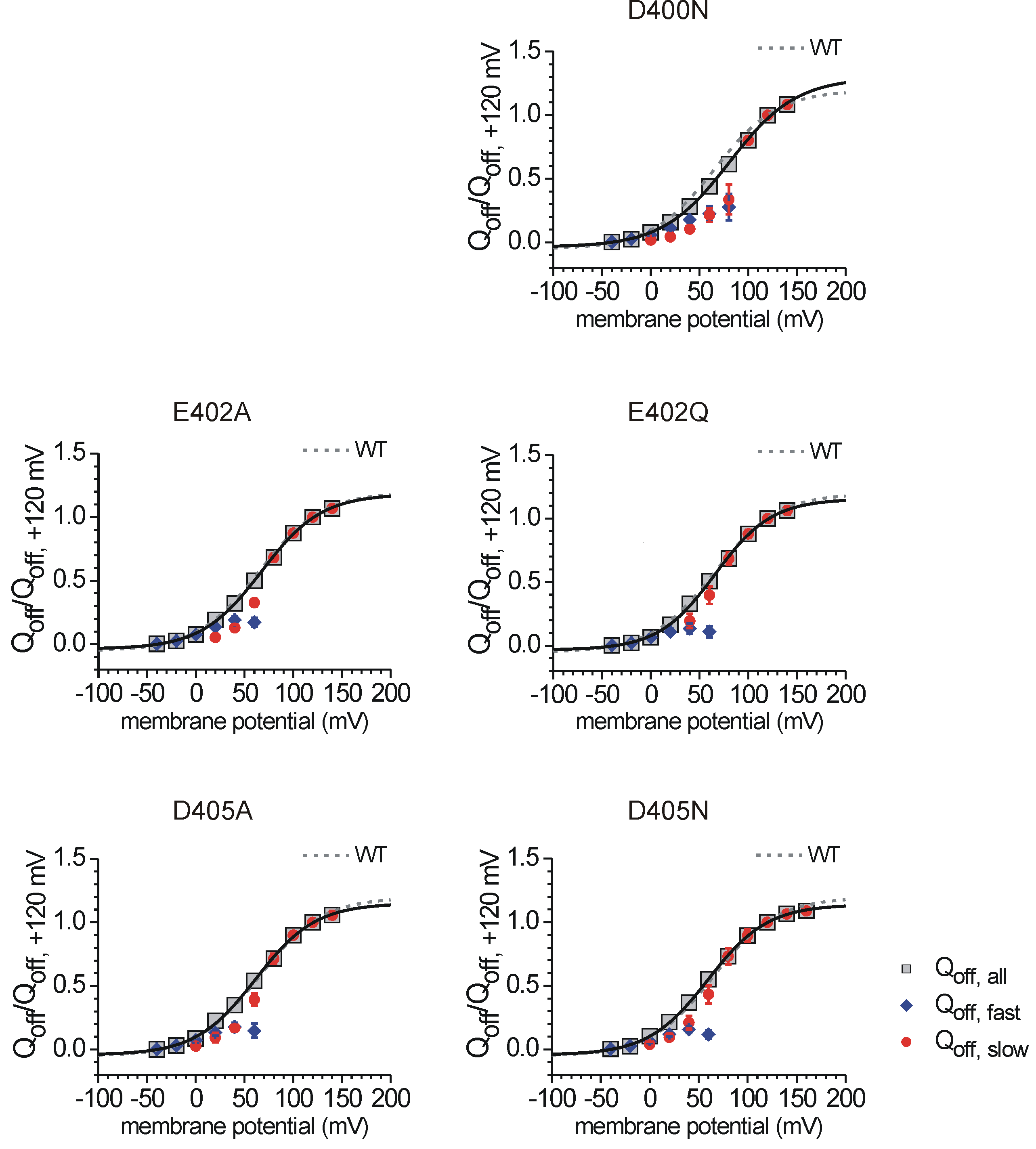

Supplement: Figure S3 — Voltage-dependence of translocated off-sensing charges for TI loop mutants. Sensing charges translocated during the off-motion of the VSD were calculated as described earlier [13]. The averaged fast (Qoff,fast in blue) and slow fraction (Qoff,slow in red) of the off-sensing charge are plotted against the membrane potential as well as the sum of both (Qoff,all in gray). The individual Qoff-values, which were obtained per oocyte, were normalized to the respective Qoff,all-value at+120 mV. Qoff,all was approximated with a Boltzmann-type function as described in Materials and Methods. Fitting parameters V0.5 and zq are given in Table S3. (TIF) [file pone.0070272.s003.tif]

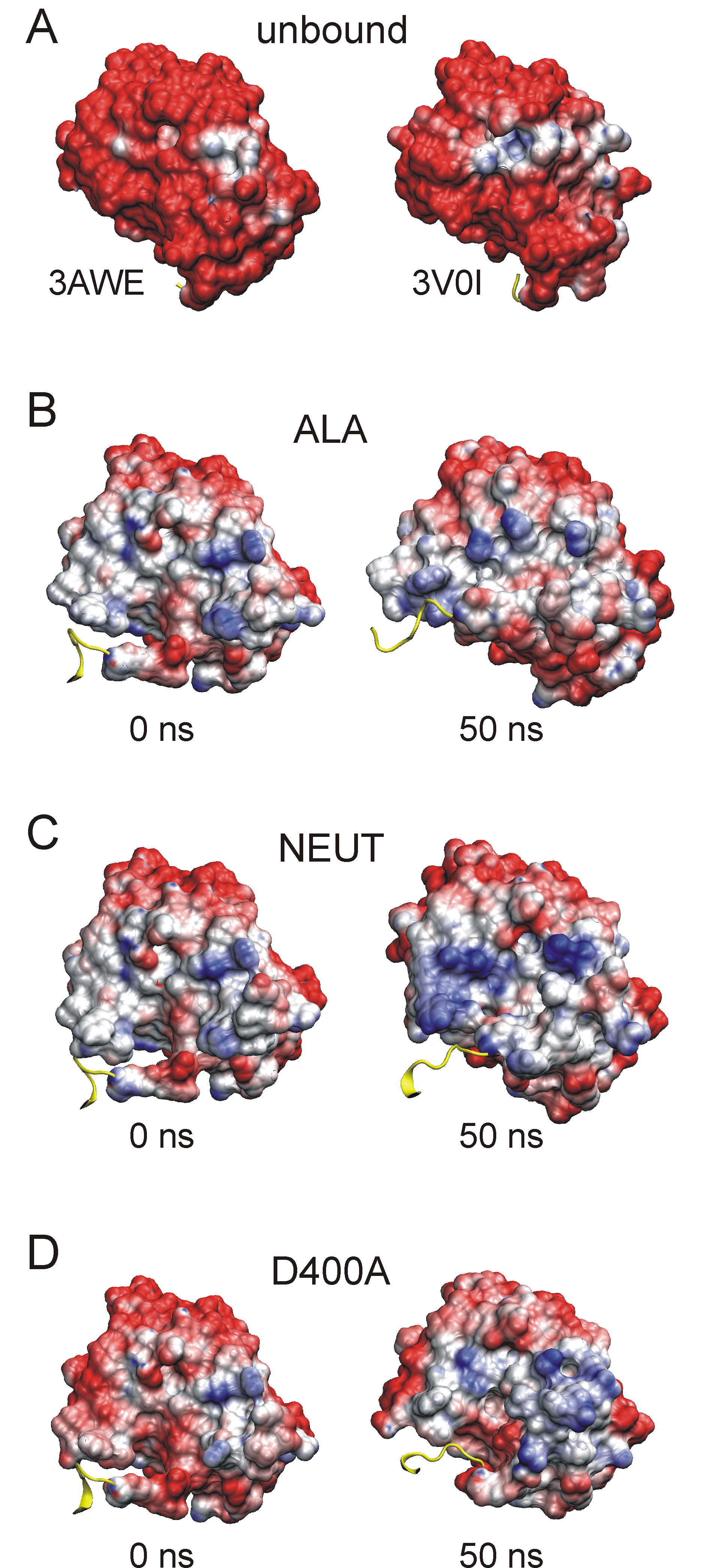

Supplement: Figure S4 — Electrostatic potential surfaces for the catalytic domain of Ci-VSP in the substrate-unbound conformation and the modeled TI loop mutants. Electrostatic potential surfaces for the phosphatase domain of Ci-VSP were calculated with the APBS tool [26]. Representations of ( A ) the unbound conformations based on the crystal structures by Matsuda et al., 2011 [15] (left panel) and Liu et al., 2012 [14] (right panel; PDB entries are given, respectively) as well as ( B–D ) for the denoted TI loop mutants before (0 ns) and after (50 ns) of MD simulation. It should be noted that the differences in electrostatic potentials between the WT (Fig. 6A) and the mutants at 0 ns is due to the reduction of the negatively charged character in the TI loop caused by the respective mutation. (TIF) [file pone.0070272.s004.tif]

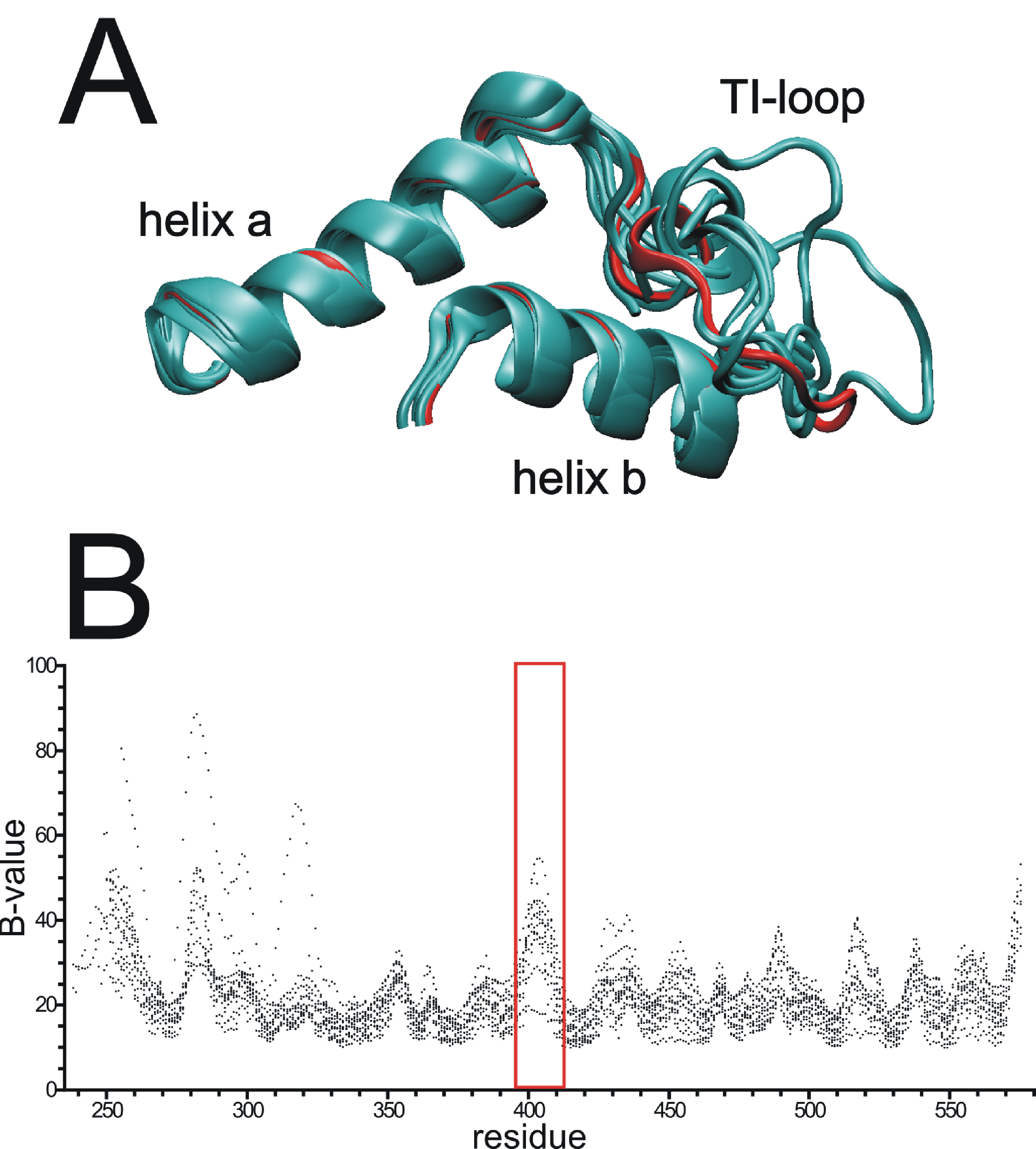

Supplement: Figure S5 — Structural alignment between the TI loop region of the initial Ci-VSP WT model and crystallographic structures. (A) The TI loop of our initial Ci-VSP WT model (red) is structurally aligned with the respective region from the crystallographic structures by Liu et al. [14] (cyan). To obtain a suitable alignment, the neighboring α-helices were superimposed, with helix a and b containing the residues T386–T399 and T412–Y429, respectively. (B) The B-values of the Cα-atoms for all residues resolved in the crystallographic structures by Liu et al. [14] are plotted. The red box marks the strongly fluctuating region of the TI loop. (TIF) [file pone.0070272.s005.tif]
