# Supplementary material for: The Linker Pivot in Ci-VSP: The Key to Unlock Catalysis
Source: PLoS One. 2013 Jul 29;8(7):e70272. doi: 10.1371/journal.pone.0070272 (PMC3726396; doi:10.1371/journal.pone.0070272)
Supplement: Table S1 — Averaged number of contacts between linker and PI(4,5)P2 residues. Number of contacts (#contacts) between the linker and PI(4,5)P2 residues averaged over the last 30 ns. Additionally, the interaction of the N-terminal (240–249) and the C-terminal (250–257) parts of the linker and PI(4,5)P2 residues is shown. As in Table 1, contacts are defined as atoms within a sphere of 3.5 Å. (DOC) [file pone.0070272.s006.doc]

| Model | #contacts | #contacts | #contacts |
| --- | --- | --- | --- |
|  | linker– PI(4,5)P_2_ | N-term linker– PI(4,5)P_2_ | C-term linker– PI(4,5)P_2_ |
| WT | 150.6 ± 13.1 | 99.3 ± 12.1 | 55.1 ± 4.5 |
| NEUT | 176.4 ± 10.5 | 88.7 ± 10.3 | 94.9 ± 4.3 |
| ALA | 75.2 ± 12.2 | 36.3 ± 5.4 | 39.0 ± 12.2 |
| D400A | 101.0 ± 14.5 | 56.7 ± 11.0 | 46.7 ± 9.1 |
