# Supplementary material for: The Linker Pivot in Ci-VSP: The Key to Unlock Catalysis
Source: PLoS One. 2013 Jul 29;8(7):e70272. doi: 10.1371/journal.pone.0070272 (PMC3726396; doi:10.1371/journal.pone.0070272)
Supplement: Table S2 — Structural changes of the linker in terms of the root-mean-square deviation. The differences between the end geometries in comparison to the WT enzyme after 50 ns are shown. Additionally, the over the last 30 ns averaged rmsd to the initial conformation is displayed. All rmsd values refer to the backbone atoms of the linker region (240 to 257). (DOC) [file pone.0070272.s007.doc]

| Model | RMSD to WT / Å | RMSD / Å |
| --- | --- | --- |
| WT | – | 2.9 ± 0.2 |
| NEUT | 3.2 | 2.1 ± 0.1 |
| ALA | 3.5 | 3.2 ± 0.2 |
| D400A | 2.6 | 2.5 ± 0.5 |
