# Supplementary material for: The Linker Pivot in Ci-VSP: The Key to Unlock Catalysis
Source: PLoS One. 2013 Jul 29;8(7):e70272. doi: 10.1371/journal.pone.0070272 (PMC3726396; doi:10.1371/journal.pone.0070272)
Supplement: Table S3 — Boltzmann-parameters for the voltage-dependent translocation of sensing charges in Ci-VSP. Amount of voltage-dependent sensing charges (Qoff,all-V-values) were determined from the transient off-currents of Ci-VSP as described in detail earlier [13]. The resulting Qoff,all-V-distributions were approximated with a Boltzmann-type function (see Materials and Methods) to determine the parameters V0.5 (midpoint potential) and zq (slope factor) which describe the voltage-dependence of the off-currents (n: numbers of independently performed measurements). (DOC) [file pone.0070272.s008.doc]

|  | V_0.5_ (mV) | z_q_ | n |
| --- | --- | --- | --- |
| WT | 64.9 ± 0.8 | 0.78 ± 0.02 | 15 |
| NEUT | 80.6 ± 8.1 | 0.56 ± 0.11 | 8 |
| ALA | 63.6 ± 0.8 | 0.90 ± 0.03 | 15 |
| D400N | 80.5 ± 2.3 | 0.74 ± 0.04 | 7 |
| E402Q | 65.8 ± 1.4 | 0.87 ± 0.04 | 14 |
| D405N | 59.4 ± 0.6 | 0.83 ± 0.02 | 13 |
| D400A | 94.0 ± 3.1 | 0.74 ± 0.06 | 6 |
| E402A | 67.3 ± 1.2 | 0.82 ± 0.05 | 21 |
| D405A | 61.4 ± 1.6 | 0.82 ± 0.05 | 16 |
